# Distinct roles of KAP1, HP1 and G9a/GLP in silencing of the two-cell-specific retrotransposon MERVL in mouse ES cells

**DOI:** 10.1186/1756-8935-6-15

**Published:** 2013-06-04

**Authors:** Irina A Maksakova, Peter J Thompson, Preeti Goyal, Steven JM Jones, Prim B Singh, Mohammad M Karimi, Matthew C Lorincz

**Affiliations:** 1 Department of Medical Genetics, Life Sciences Institute, 2350 Health Sciences Mall, University of British Columbia, Vancouver, British Columbia V6T 1Z3, Canada; 2 British Columbia Cancer Agency, Genome Sciences Centre, 675 West 10th Avenue, Vancouver, British Columbia V5Z 4S6, Canada; 3 Fächereverbund Anatomie, Institut für Zell and Neurobiologie, Charite - Universitätsmedizin, Charitéplatz 1, 10117, Berlin, Germany

**Keywords:** G9a, GLP, HP1, KAP1, MERVL, ERV, Chimeric transcripts, Preimplantation, H3K9me2, mESC

## Abstract

**Background:**

In mouse embryonic stem cells (mESCs), transcriptional silencing of numerous class I and II endogenous retroviruses (ERVs), including IAP, ETn and MMERVK10C, is dependent upon the H3K9 methyltransferase (KMTase) SETDB1/ESET and its binding partner KAP1/TRIM28. In contrast, the H3K9 KMTases G9a and GLP and HP1 proteins are dispensable for this process. Intriguingly, MERVL retroelements are actively transcribed exclusively in the two-cell (2C) embryo, but the molecular basis of silencing of these class III ERVs at later developmental stages has not been systematically addressed.

**Results:**

Here, we characterized the roles of these chromatin factors in MERVL silencing in mESCs. While MMERVK10C and IAP ERVs are bound by SETDB1 and KAP1 and are induced following their deletion, MERVL ERVs show relatively low levels of SETDB1 and KAP1 binding and are upregulated exclusively following KAP1 depletion, indicating that KAP1 influences MERVL expression independent of SETDB1. In contrast to class I and class II ERVs, MERVL and MERVL LTR-driven genic transcripts are also upregulated following depletion of G9a or GLP, and G9a binds directly to these ERVs. Consistent with a direct role for H3K9me2 in MERVL repression, these elements are highly enriched for G9a-dependent H3K9me2, and catalytically active G9a is required for silencing of MERVL LTR-driven transcripts. MERVL is also derepressed in HP1α and HP1β KO ESCs. However, like KAP1, HP1α and HP1β are only modestly enriched at MERVL relative to IAP LTRs. Intriguingly, as recently shown for KAP1, RYBP, LSD1 and G9a-deficient mESCs, many genes normally expressed in the 2C embryo are also induced in HP1 KO mESCs, revealing that aberrant expression of a subset of 2C-specific genes is a common feature in each of these KO lines.

**Conclusions:**

Our results indicate that G9a and GLP, which are not required for silencing of class I and II ERVs, are recruited to MERVL elements and play a direct role in silencing of these class III ERVs, dependent upon G9a catalytic activity. In contrast, induction of MERVL expression in KAP1, HP1α and HP1β KO ESCs may occur predominantly as a consequence of indirect effects, in association with activation of a subset of 2C-specific genes.

## Background

Endogenous retrovirus-like sequences (ERVs) are fossils of ancient retroviral integrations into the mammalian germline. Multiple independent colonization events have led to the accumulation of over 400 different ERV families with defined transcriptional patterns, often limited to specific developmental stages and cell types. Based on the similarity of their reverse transcriptase genes, ERVs are grouped into three classes: I, II and III, most closely related to exogenous gammaretroviruses, betaretroviruses and spumaretroviruses, respectively [1]. Most ERVs in each class are no longer capable of transcription and/or retrotransposition due to the accumulation of mutations and/or efficient targeting by host silencing mechanisms that act at various stages of the viral life cycle [2]. Nevertheless, many ERVs possess functional regulatory sequences that direct transcription at specific developmental stages and/or in specific tissues. A number of ERV subfamilies are particularly active early in embryogenesis [3], likely due to selection for expression and in turn retrotransposition at those stages in development that maximize the likelihood of germline transmission.

The class III MT subfamily of MaLR retrotransposons, for example, which comprise less than 5% of the mouse genome [4], account for 13% of all transcripts in the fully grown oocyte [3]. The related class III ERV MERVL/MuERV-L (mouse ERV with a leucine tRNA primer-binding site), of which there are 656 full-length copies and 37,172 solitary long terminal repeats (LTRs) in the C57BL/6 genome (based on Repeatmasker analysis), are among the first sequences to be transcribed in the early two-cell (2C) embryo and account for nearly 4% of the mouse transcriptome at the 2C stage [3,5-7]. Class II intracisternal A-type particle (IAP) ERVs on the other hand, of which there are well over 600 full-length copies in the mouse genome, only account for 0.6% of the 2C transcriptome [3]. Intriguingly, MERVL expression may be essential for development beyond the four-cell stage [5], perhaps due to the exaptation of ERV LTRs as promoters for essential genes [8,9]. Indeed, MERVL-driven genic transcripts are abundant at the 2C stage [3,10] and in mouse embryonic stem cells (mESCs) that show 2C-like features [11,12]. Given that such transcripts are not detectable at later developmental stages, it is likely that the LTR promoters of such ‘chimeric’ genes are regulated by the same epigenetic mechanisms that govern the ERVs from which they are derived.

To minimize the generally deleterious effects associated with retrotransposition, a number of pathways have evolved to inhibit transcription of ERVs. DNA methylation, mediated by the *de novo* methyltransferases DNMT3A and DNMT3B and the maintenance methyltransferase DNMT1, plays a critical role in proviral silencing in somatic tissues [13-15] including fibroblasts [16,17], as well as in late germline development [18,19]. Surprisingly however, while class I and II ERVs show broad DNA demethylation in G9a and GLP knockout (KO) mESCs [20], neither of these lysine methyltransferases (KMTases), which dimethylate lysine 9 of histone H3 (H3K9), are required for silencing of these ERVs. Furthermore, while IAP transcript levels are elevated in DNMT1-deficient relative to wild-type (wt) mESCs [21,22], this difference increases dramatically in mESCs cultured in the absence of leukemia inhibitory factor (LIF) [21], indicating that a DNA methylation-independent mechanism may also operate in undifferentiated ESCs to silence such ERVs.

Indeed, we recently reported that in mESCs, numerous class I and II ERVs, including MMERVK10C, MusD and ETn elements, are de-depressed in the absence of the H3K9 KMTase SETDB1/ESET, while IAP elements show the highest level of activation in the absence of both DNMT1 and SETDB1 [23,24]. Furthermore, robust silencing of each of these ERVs is dependent upon H3K9me3 deposited by SETDB1 [24,25] and the corepressor KAP1/TRIM28/TIF1-β, which directly interacts with the KAP1 interaction domain (KID) of SETDB1 [26]. As KAP1 can directly interact with any one of several hundred Krüppel-associated box zinc finger proteins (KRAB-ZFPs), we and others have proposed that the KAP1/SETDB1 complex may generally be recruited to ERVs by KRAB-ZFPs that recognize specific ERV sequences [23,24,27-30]. Curiously however, while class III MERVL elements are also upregulated in KAP1 KO mESCs [28], we observed only modest upregulation of these ERVs (approximately 2-fold) in SETDB1 KO mESCs [23].

Heterochromatin protein 1 (HP1) proteins, which encode chromo- and chromoshadow domains, function in both structural and gene regulatory pathways in eukaryotes [31-33]. These H3K9me ‘readers’ modulate gene expression in part through binding to H3K9me2/3 via the chromodomain [34-39]. In addition, HP1 proteins directly interact with the PxVxL motif of KAP1 via their chromoshadow domain independent of H3K9 methylation state [29,40-42]. Intriguingly, this interaction is required for transcriptional silencing of reporter genes [34,43] as well as of the nonimprinted *Mest* allele in embryonal carcinoma cells [42]. Surprisingly however, we recently showed that depletion of HP1α (encoded by *Cbx5*), HP1β (encoded by *Cbx1*) and/or HP1γ (encoded by *Cbx3*), alone or in combination, does not lead to derepression of class I or class II ERVs in mESCs [25], raising the question: do HP1 proteins play a role in repression of class III ERVs?

Here, using genetic knockouts and/or RNAi, we analyzed the roles of KAP1, HP1α, HP1β as well as G9a and GLP, in transcriptional silencing of MERVL elements in mESCs. Our results indicate that MERVL expression is induced as a consequence of both direct and indirect effects, the former due to loss of H3K9me2 and the latter in association with derepression of genes normally expressed at the 2C stage.

## Results

### Class II ERVs are upregulated in KAP1- and SETDB1-deficient cells, while MERVL ERVs are upregulated exclusively in KAP1-deficient cells

**Figure 1:**
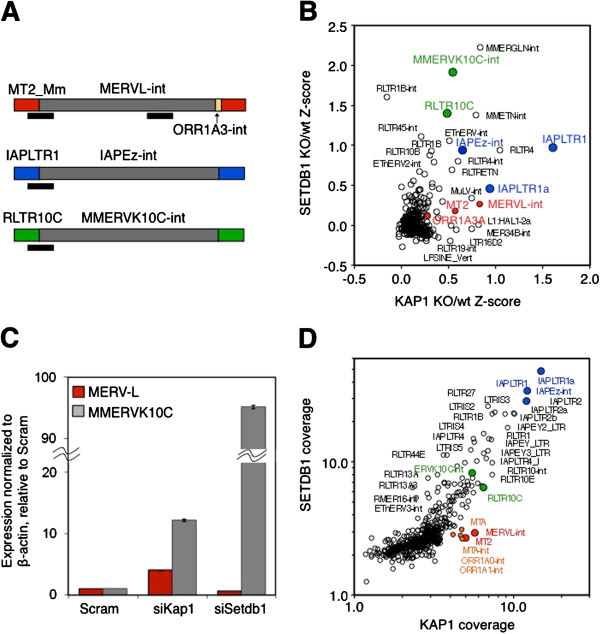
**MERVL ERVs are derepressed upon KAP1 but not SETDB1 depletion, while MMERVK10C and IAP ERVs are upregulated following depletion of both.** (**A**) Repbase annotations of the LTR and internal regions of full-length MERVL, IAPEz and MMERVK10C elements are shown. Black bars indicate qPCR amplicons for LTRs of each family of element and the internal *pol* gene region for MERVL. (**B**) Deregulation of transposable element families in KAP1 and SETDB1 KO mESCs. RNA-seq data for SETDB1 [23] and KAP1 [28] KO lines and the corresponding wt cell lines were used to calculate Z-score values for all annotated retroelements and plotted as shown. (**C**) KAP1 and SETDB1 were depleted by RNAi in wt TT2 mESCs, and reactivation of MERVL and MMERVK10C elements was determined by qRT-PCR. Mean expression (+/-SD) of each ERV (normalized to β-actin) relative to a scrambled siRNA pool (Scram) is shown for three technical replicates. (**D**) MERVL is among a small group of transposable elements bound by KAP1 but not SETDB1. RPKM (*10) values generated from published ChIP-seq data for KAP1 [46] and SETDB1 [47] were plotted for all retroelements. Numerous class I and class II ERVs, including IAP subfamilies are enriched for both proteins, which are generally strongly correlated. MERVL elements are modestly enriched for KAP1, but show relatively low levels of SETDB1 coverage. ChIP-seq, chromatin immunoprecipitation sequencing; RPKM, reads per kilobase per million mapped reads.

We recently showed that silencing of many class I and II ERV families is maintained in mESCs by SETDB1-mediated deposition of H3K9me3 [23,24]. Consistent with a previous report [28], we also found that the SETDB1-associated corepressor KAP1 is required for repression of many of the same ERVs [24], supporting an essential role for the KAP1-SETDB1 complex [44] in proviral repression. Curiously however, while MERVL elements were also reported to be dramatically upregulated in KAP1 KO mESCs [11,28], our genome-wide analysis revealed minimal derepression of this class III ERV family in SETDB1 KO mESCs [23]. Reanalysis of RNA-sequencing (RNA-seq) data from SETDB1 KO [23] and KAP1 KO mESCs [28], confirmed that many class I and II families are derepressed in both cell lines (Figure 1A-B). IAPEz and MMERVK10C (a close relative of IAP [45]) ERVs, for example, are upregulated 14- and 100-fold, respectively, in the SETDB1 KO, versus 24- and 26-fold, respectively, in the KAP1 KO. In contrast, while MERVL elements, composed of three annotations in the UCSC genome browser: MT2_Mm (LTR), MERVL-int (internal region) and ORR1A3-int (Figure 1A), are upregulated 27-fold in the KAP1 KO, they are upregulated less than 3-fold in the SETDB1 KO (Figure 1B). Knockdown (KD) of *Setdb1* or *Kap1* by RNAi followed by qRT-PCR yielded similar results (Figure 1C), ruling out the possibility that the distinct phenotypes observed in SETDB1 versus KAP1 KO mESCs are due to differences in genetic background. Thus, while silencing of class III MERVL elements is indeed dependent upon KAP1, depletion of SETDB1 has a relatively modest effect on MERVL expression, revealing that KAP1 plays a role in silencing of this ERV subfamily independent of SETDB1.

**Figure 2:**
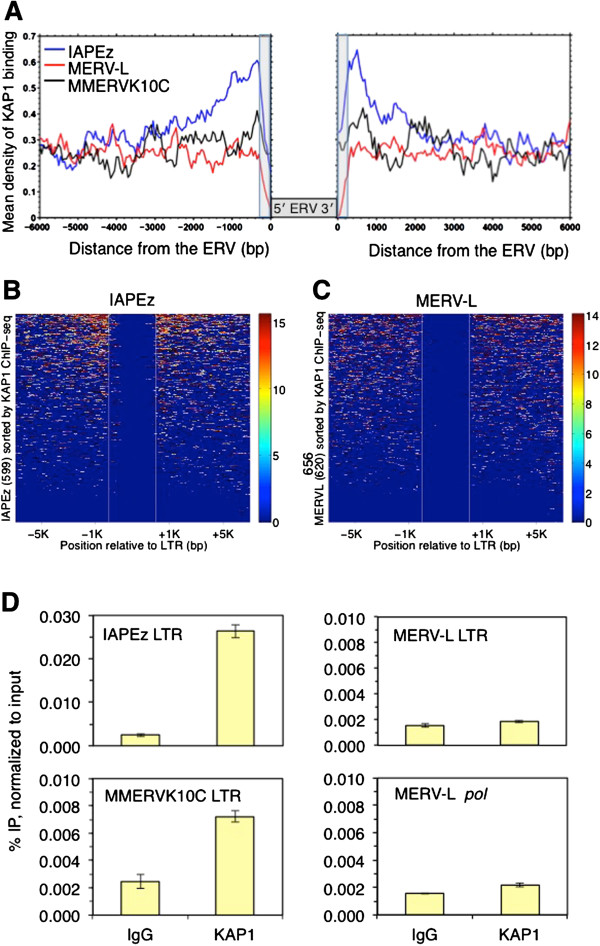
**Unique regions flanking IAPEz but not MERVL elements are highly enriched in KAP1.** (**A**) Profiling of KAP1 in the flanking sequence of all full-length IAPEz, MERVL and MMERVK10C ERVs. KAP1 ChIP-seq reads [46] from wt mESCs were aligned to the mouse genome (mm9), and the density profile of unique reads mapping to the 6 kb regions flanking all annotated intact MERVL (656), MMERVK10C (298) and IAPEz (599) elements, was plotted as shown. (**B-C**) Heat maps of KAP1 enrichment in the genomic regions flanking 599 IAPEz and 656 MERVL elements in wt mESCs. KAP1 ChIP-seq reads [46] were aligned to the mouse genome (mm9), and the density of uniquely aligned reads, mapping to the 6 kb regions flanking all intact ERVs of the specified families, was plotted. Reads extending into the ERV are due to *in silico* extension of aligned reads by 300 bp. (**D**) ChIP and qPCR analysis of KAP1 in TT2 wt mESCs at the LTRs of IAPEz, MMERVK10C and MERVL, as well as the MERVL *pol* internal region. IgG, negative control IP. Data are mean enrichment from three technical replicates as a percentage of the input chromatin and error bars represent SD. IgG, immunoglobulin G; IP, immunoprecipitation; SD, standard deviation.

To determine whether MERVL elements are bound by KAP1 and/or SETDB1, we performed meta-analysis of published KAP1 and SETDB1 chromatin immunoprecipitation sequencing (ChIP-seq) datasets [46,47]. Numerous class I and class II ERVs, including IAP subfamilies, show significant enrichment of both factors and a strong positive correlation between the two (Figure 1D), consistent with our previous observations that these ERV families are marked by H3K9me3 in a SETDB1-dependent manner [23]. In contrast, MERVL is one of a small group of ERVs showing no detectable enrichment of SETDB1 and low levels of cumulative KAP1 binding relative to most SETDB1-bound class I and II ERVs (Figure 1D). While class III MaLR ERVs ORR1A and MTA are enriched for KAP1 at levels similar to MERVL, only MERVL is upregulated in KAP1 KO mESCs. To determine whether KAP1 binding at MERVL elements is associated with enrichment of KAP1 in the unique regions flanking these proviruses, we analyzed KAP1 enrichment in the nonrepetitive sequences flanking all 656 full-length MERVL, 298 MMERVK10C and 599 IAPEz elements. As shown previously for H3K9me3 [25,48], KAP1 binding is clearly higher in the immediate flanks of IAP elements when analyzed in aggregate, decreasing to background levels with increasing distance to the provirus (Figure 2A). MMERVK10C elements also show enrichment in proximal versus distal flanking regions, although to a lesser extent. This pattern is common to multiple individual IAP elements (Figure 2B); consistent with the hypothesis that KAP1 binds directly to this class II ERV and spreads into neighboring genomic regions. In contrast, enrichment of KAP1 is not detected at the flanks of MERVL elements when analyzed in aggregate (Figure 2A) and is sparsely detected at the flanks of only a small fraction of MERVL elements when analyzed individually (Figure 2C). Furthermore, in contrast to the sequences flanking individual IAP elements, enrichment is evenly distributed across the 6 kb regions flanking the few individual MERVL elements that show KAP1 binding in their flanks, indicating that the low level of KAP1 enrichment observed within these elements may reflect the chromatin state of the locus/integration site, rather than direct recruitment of KAP1 to these elements. Alternatively, in the absence of SETDB1-mediated H3K9me3 deposition, spreading of KAP1 into the regions flanking MERVL elements may not occur, leading to a relatively low level of focal KAP1 enrichment within the regulatory region of these ERVs. As an alternative approach to quantify KAP1 enrichment specifically within MERVL elements, we conducted ChIP using a KAP1-specific antibody. Consistent with the ChIP-seq data, KAP1 enrichment was detected at IAP and to a lesser extent at MMERVK10C LTRs (Figure 2D). In contrast, no enrichment was detected at LTR or *pol* internal regions of MERVL, using primers that detect 519 and 637 elements, respectively, as determined by *in silico* PCR. While we cannot rule out the possibility that relatively weak, localized binding of KAP1 to MERVL LTRs plays a direct, SETDB1-independent role in silencing of these elements, SETDB1/H3K9me3 is routinely observed at repressed native loci and transgenes bound by KAP1 [23,26,29]. Furthermore, KAP1 mutants that cannot interact with SETDB1 are defective in silencing of KAP1-bound transgenes [26]. Thus, our observations are also consistent with the hypothesis that derepression of MERVL elements in KAP1-deficient mESCs occurs as a consequence of indirect effects.

### MERVL elements and chimeric transcripts are upregulated in G9a and GLP KO mESCs

**Figure 3:**
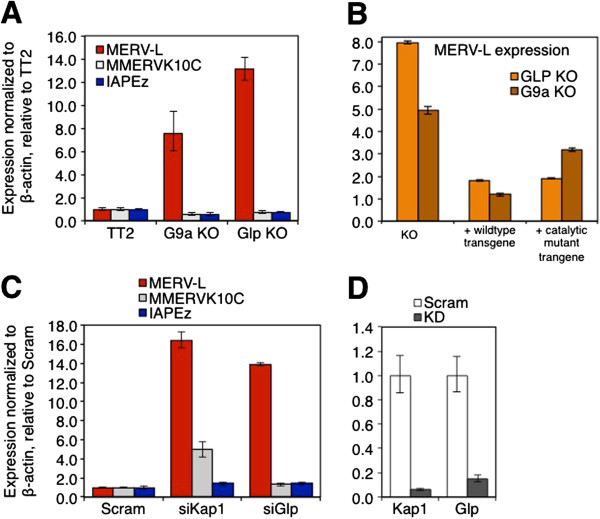
**MERVL ERVs are derepressed in G9a- and GLP-deficient mESCs and MERVL silencing is dependent on G9a catalytic activity.** (**A**) Upregulation of MERVL in G9a and GLP KO mESCs. Expression of MERVL, MMERVK10C and IAPEz ERVs in TT2 wt, G9a and GLP KO mESCs was analyzed by qRT-PCR. Mean (+/-SD) expression levels relative to the wt line for three technical replicates (normalized to β-actin) are shown. (**B**) Catalytic activity of G9a but not GLP is required for MERVL silencing. G9a or GLP KO mESCs stably expressing wt or catalytic mutant G9a (C1168A) or GLP (C1201A) [50] transgenes, respectively, were assessed for MERVL expression by qRT-PCR, as described above. (**C**) MERVL but not MMERVK10C or IAPEz ERVs are upregulated upon KD of *Glp*. Relative expression of ERVs was determined by qRT-PCR, as described above. (**D**) Efficiency of each KD was determined by qRT-PCR with primers specific for *Kap1* and *Glp*, as described above. SD, standard deviation.

While we have shown previously that silencing of class I and II ERVs is not dependent upon G9a [20], we did not address whether this H3K9me1/2 KMTase plays a role in repression of class III ERVs. As MERVL elements were recently shown to be marked by H3K9me2 [11], we next tested whether G9a and the closely related KMTase GLP, which form a heterodimer [49], are required for silencing of MERVL ERVs. Quantitative RT-PCR analysis of G9a KO and GLP KO mESCs revealed that MERVL elements are upregulated approximately 8-fold and approximately 13-fold respectively, relative to their parent line TT2 (Figure 3A). In contrast, MMERVK10C, similar to IAP ERVs (Figure 3A and [20]), showed no change in expression in either the G9a or GLP KO lines. Analysis of G9a or GLP KO mESCs stably expressing wt or catalytic mutants of G9a (G4, C1168A) or GLP (L4, C1201A), respectively [50], revealed that MERVL silencing is dependent only upon catalytically active G9a (Figure 3B). This result may be explained by the fact that while G4 and L4 catalytic mutants form heteromeric complexes with wt GLP and G9a, respectively, H3K9me2 levels are restored only in the GLP KO line rescued with the L4 catalytic mutant [50], implicating G9a as the critical H3K9 KMTase in the context of the G9a/GLP heterodimer. Transient depletion of GLP also disrupts MERVL silencing (siRNAs directed against *G9a* did not yield efficient depletion of *G9a* mRNA), with a 14-fold increase in MERVL expression observed 4 days post siRNA transfection (Figure 3C-D). In contrast, as expected, no increase in MMERVK10C or IAPEz expression was observed following KD of *Glp*. While MERVL expression was induced approximately 16-fold following KAP1 KD, MMERVK10C was induced only approximately 5-fold in this experiment and IAPEz – only 1.5-fold, likely due to DNA methylation-mediated repression [23] and/or insufficient depletion of the protein. Importantly, KAP1 and LSD1 protein levels are not reduced in GLP or G9a KO mESCs (Additional file 1: Figure S1A), indicating that derepression of MERVL elements in these cells is not due to destabilization of these proteins, which were previously implicated in MERVL silencing [11,28]. Furthermore, while interactions between KAP1 and HP1β and G9a and HP1β were clearly detected by co-immunoprecipitation (co-IP), as reported previously [40,41,51], no interaction between KAP1 and G9a was detected under the same conditions (Additional file 1: Figure S1B), indicating that G9a is unlikely to regulate MERVL elements via direct interaction with KAP1.

LTRs that are derepressed in mESCs deficient in SETDB1 or the H3K4me1/2 demethylase LSD1/KDM1A can function as alternative promoters for downstream genes [11,23], and the specific ERV families upregulated in these KOs contribute to the majority of such aberrantly expressed chimeric transcripts. To determine whether naturally occurring chimeric transcripts that initiate in MERVL/MT2 LTRs and splice to downstream genic exons are also upregulated in G9a- or GLP-depleted mESCs, we first identified all protein-coding genes represented in both RefSeq and ENSEMBL databases with an MT2 LTR as the annotated exon 1 (Additional file 2). Among the 43 genes on this list, 10 and 6 were upregulated >4-fold in KAP1 and G9a KO mESCs, respectively, relative to their wt parent lines, as determined by meta-analysis of previously published RNA-seq data [12,28]. Strikingly, of the six upregulated genes in the G9a KO line, five are also upregulated in the KAP1 KO line. The MT2B LTR-driven gene *Zfp352*, which is upregulated dramatically in both of these KO lines, is induced approximately 80-fold in *Glp* KD mESCs, as determined by qRT-PCR analysis (Additional file 1: Figure S1C), confirming that at least a subset of MERVL LTR-driven genic transcripts are silenced by G9a and GLP. Together, these data demonstrate that the G9a/GLP complex is not only required for silencing of intact MERVL elements but also plays a critical role in silencing of a subset of the annotated genes that initiate in MERVL LTRs.

### MERVL elements are direct targets of the G9a/GLP H3K9 KMTase complex

**Figure 4:**
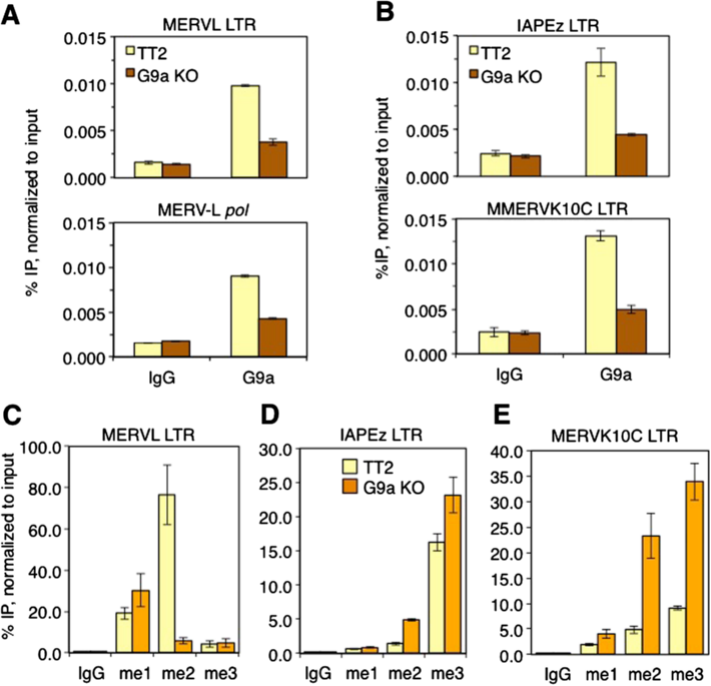
**G9a is bound at MERVL and H3K9me2 enrichment at MERVL elements is dependent on G9a.** (**A-B**) ChIP and qPCR of G9a in TT2 wt and G9a KO mESCs at LTR of MERVL and MERVL internal region and LTRs of IAPEz and MMERVK10C. IgG, negative control IP. Data are mean enrichment from three technical replicates as a percentage of the input chromatin and error bars represent SD. (**C-E**) N-ChIP and qPCR was performed for H3K9me1 (me1), H3K9me2 (me2), H3K9me3 (me3) and IgG as a negative control in TT2 wt and G9a KO mESCs at the LTRs of MERVL, IAPEz and MMERVK10C. Data are mean enrichment (+/-SD) for three technical replicates normalized to input. IgG, immunoglobulin G; IP, immunoprecipitation; SD, standard deviation.

To determine whether G9a is directly bound at MERVL elements, we performed cross-linked ChIP in wt and G9a KO mESCs using a G9a-specific antibody. In contrast to KAP1, G9a was specifically enriched in the LTR and *pol* gene regions of MERVL (Figure 4A) and was also detected at IAPEz and MMERVK10C LTRs (Figure 4B). Importantly, G9a was depleted at these regions in the G9a KO line, confirming the specificity of this antibody. To determine whether MERVL elements are marked by H3K9me2 in a G9a-dependent manner, we analyzed all three states of H3K9 methylation in wt and G9a KO mESCs by native ChIP (N-ChIP). H3K9me2 is highly enriched at the MERVL 5′LTR/promoter region in wt mESCs and reduced to near background levels in G9a KO mESCs (Figure 4C). In contrast, the 5′LTR/promoter regions of MMERVK10C and IAPEz ERVs show relatively low but clearly detectable levels of H3K9me2 in wt mESCs (Figure 4D-E), as shown previously [20]. The converse is true for H3K9me3, consistent with our previous observations that class II ERVs, including IAPEz and MMERVK10C, are directly regulated by SETDB1 [23,24] and the results presented above, which reveal that MERVL elements are directly regulated by G9a and GLP. Taken together, these results indicate that the G9a/GLP heterodimer plays a direct role in silencing of MERVL elements.

### MERVL and MERVL-promoted chimeric genes are upregulated in HP1α and HP1β KO mESCs

**Figure 5:**
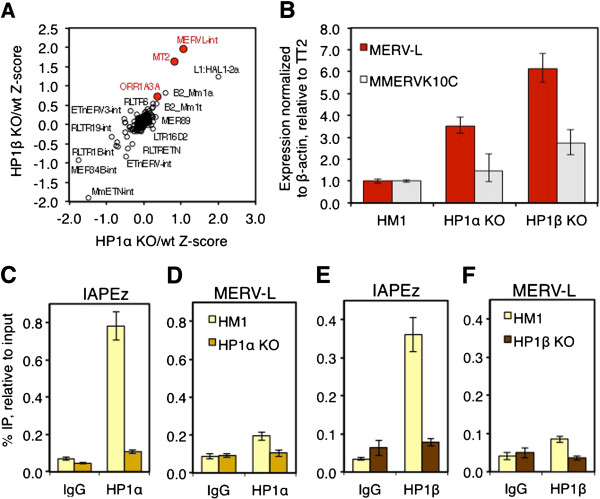
**MERVL ERVs are derepressed in HP1α and HP1β KO mESCs but HP1α and HP1β show relatively low enrichment at these ERVs.** (**A**) RNA-seq analysis of retroelement expression in HP1α and HP1β KO mESCs. RNA-seq data for HP1α and HP1β KO mESCs and the HM1 parent line was generated, and Z-scores were calculated for all retroelements and plotted. Note that MERVL elements show relatively high levels of derepression in both KO lines. (**B**) MERVL elements are upregulated in HP1α and HP1β KO mESCs. Expression of MERVL, IAP and MMERVK10C ERVs was analyzed by qRT-PCR in HM1 wt, HP1α and HP1β KO lines. Mean (+/-SD) expression levels (normalized to β-actin) are shown, relative to the wt line for three technical replicates. (**C-F**) IAPEz elements show significantly higher levels of HP1α and HP1β enrichment than do MERVL elements. Cross-linked ChIP was performed with antibodies specific for HP1α or HP1β in HM1 and the corresponding KO cell line. IgG was used as a negative control. The level of enrichment for each IP was determined by qPCR, and the mean and standard deviation of three technical replicates are shown for each experiment. IgG, immunoglobulin G; IP, immunoprecipitation; SD, standard deviation.

HP1 proteins are thought to play an important role in transcriptional silencing via binding to methylated H3K9, leading to chromatin compaction and heterochromatin spreading [29,35,37]. While these corepressors have highest affinity for H3K9me3, they can also bind H3K9me2 *in vitro* and have been reported to directly interact with both KAP1 and the G9a/GLP complex [35,40,41,51]. To determine whether HP1 proteins are required for silencing of MERVL elements, we generated RNA-seq data for mESCs deficient in HP1α or HP1β and the wt parent line HM1 (described in [25]). Intriguingly, MERVL elements are among the most highly upregulated retrotransposons in both HP1α and HP1β KO lines (Figure 5A), showing increases in expression of 6-fold and 11-fold, respectively, relative to the parent HM1 mESC line. These elements are also among the few ERVs upregulated in both HP1 and KAP1 KO mESCs (Additional file 1: Figure S2A). Consistent with the RNA-seq data, qRT-PCR analysis revealed that MERVL elements are upregulated approximately 4-fold and approximately 6-fold in HP1α and HP1β KO mESCs, respectively (Figure 5B). Importantly, analysis of RNA-seq reads that uniquely align to specific full-length MERVL elements, of which there are 656 genomic copies, reveals that the same elements are de-repressed in the HP1α KO and HP1β KO lines (Additional file 1: Figure S2B).

To determine whether genes that initiate in MERVL LTRs are also derepressed in HP1-depleted ESCs, we calculated the expression levels of each of the 43 annotated MT2-initiated genic transcripts. Four and six of these genes were upregulated >4-fold in the HP1α and HP1β KO lines, respectively, relative to the wt parent line HM1 (Additional file 2). Strikingly, four of the genes in each case were also among the genes upregulated in the KAP1 and G9a KO lines. *Zfp352* for example, showed an increase in expression of 14- and 28-fold in the HP1α and HP1β KO lines, respectively. To determine whether additional unannotated genic transcripts (that is transcripts not present in the RefSeq or ENSEMBL databases) initiate in MERVL/MT2 LTRs in HP1 mutant cells, we identified all transcripts in our paired-end RNA-seq data in which one of the mate pairs aligns to an LTR element and the other to an annotated genic exon and scored all genes having >5 unique support reads for each in HM1 (wt), HP1α and HP1β KO mESCs. While ERVs of all three classes are represented among constitutively expressed chimeric transcripts in wt mESCs (Additional file 1: Figure S2C), the number of MERVL LTR-driven chimeric genes is higher in HP1 KO mESCs, with MT2 transcripts contributing 19% and 28% of all chimeric transcripts in the HP1α (15 of 78 total) and HP1β KO (28 of 102 total) lines, respectively, compared to 12% (5 out of 43) in wt cells (Additional file 1: Figure S2C). Together, these data demonstrate that HP1α and HP1β are required for silencing of MERVL elements as well as genic transcripts that initiate in MERVL LTRs.

### HP1α and HP1β are only modestly enriched at the 5′LTR of MERVL

To determine whether HP1 proteins are enriched specifically at MERVL LTRs, HP1α and HP1β binding was analyzed via ChIP-qPCR using chromatin extracts isolated from HM1 wt and HP1 KO mESCs. An approximately 8-fold higher level of enrichment of HP1α was detected in the 5′LTR region of IAPEz elements relative to the immunoglobulin G (IgG) control immunoprecipitation (IP), consistent with our previous observations [24] (Figure 5C). This enrichment was lost in HP1α KO mESCs, confirming the specificity of the HP1α antibody. Importantly, HP1α binding was also reduced at IAPEz elements and at the promoter region of the single-copy imprinted gene *Mest* following KD of *Kap1* (Additional file 1: Figure S2D-E), confirming that HP1α binding to these loci is KAP1-dependent [42]. In contrast, the level of HP1α enrichment observed in the 5′LTR region of MERVL elements was only approximately 2-fold higher than the IgG control IP (Figure 5D), despite comparable numbers of genomic copies recognized by the MERVL LTR (519 elements) and IAP LTR (638 elements) primer pairs, which were also used in the analysis of H3K9 methylation state described above. A similar pattern was observed for HP1β, which is enriched approximately 7-fold and 2-fold at IAPEz and MERVL LTRs, respectively (Figure 5E-F). Thus, while MERVL elements are upregulated in HP1α and HP1β KO mESCs, these KAP1-interacting factors, like KAP1 itself, are enriched at relatively low levels at MERVL LTRs.

### 2C-specific genes are induced in KAP1, G9a, HP1α and HP1β, KO mESCs

**Figure 6:**
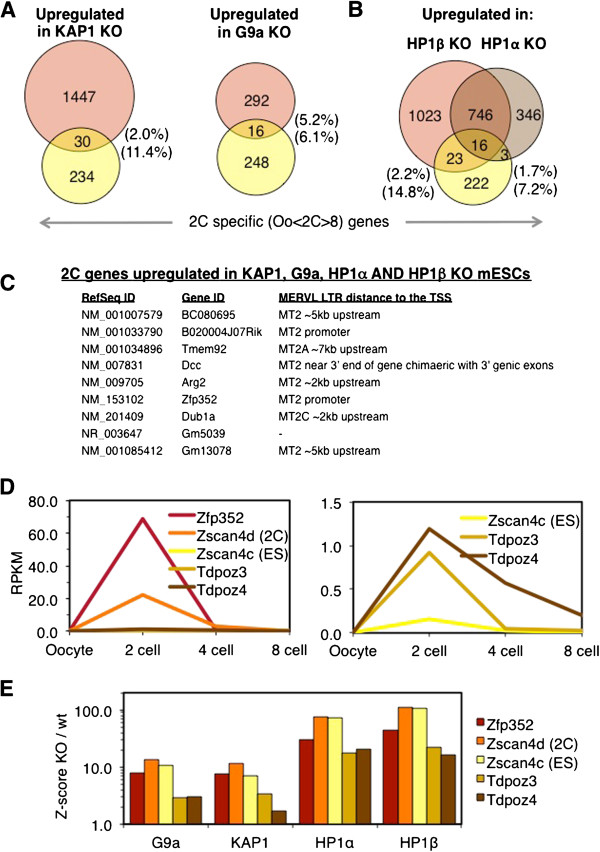
**Two-cell embryo-specific genes are induced in HP1α, HP1β, KAP1 and G9a KO mESCs.** A list of two-cell (2C) specific genes was produced from single blastomere expression data [52] by identifying genes expressed at levels 4-fold higher at the 2C stage than the oocyte or 8C stages (Oo < 2C > 8C). (**A-B**) Venn diagrams illustrating the overlap between this gene list and the list of genes upregulated at least 4-fold in KAP1, G9a [12,28], HP1α or HP1β KO mESCs are shown. The percentage of genes upregulated in the KO that are also 2C-specific is displayed above, while the percentage of 2C-specific genes that are also upregulated in the KO are presented below. (**C**) The nine genes upregulated in all four KO lines are listed, along with the distance of the nearest MERVL LTR (MT2) to the transcription start site (TSS). (**D**) Confirmation of the expression pattern of previously identified 2C-specific genes. RPKM values, derived by division of reads per million (RPM) values from RNA-seq data generated from pooled single blastomeres [52] by transcript length, are presented for *Zfp352, Zscan4d, Zscan4c, Tdpoz3* and *Tdpoz4* genes. (**E**) Expression levels of these 2C-specific genes was determined in G9a, KAP1, HP1α and HP1β KO mESCs as well as their wt parent lines using our RNA-seq data (HP1α and HP1β) or previously published RNA-seq data (G9a and KAP1 [12,28]), and Z-score values (see Materials and Methods) for each are presented.

Intriguingly, an increase in the percentage of cells permissive for MERVL expression and upregulation of numerous transcripts normally expressed at the 2C stage was recently reported for KAP1-, LSD1- and G9a-deficient mESCs [12]. These observations raise the possibility that MERVL elements are derepressed in these KO lines at least in part as a result of indirect effects of establishment of a cellular fate permissive for MERVL transcription. To determine whether 2C-specific genes are also upregulated in HP1α and/or HP1β KO mESCs, we first generated a list of genes expressed specifically at this embryonic stage, based on published RNA-seq data from single blastomeres [52]. We identified genes that are expressed at levels at least 4-fold higher at the 2C stage than the oocyte or eight-cell stages (oocyte <2C >8C). Of 264 such 2C-‘specific’ genes, 11% and 6% were upregulated relative to their wt parent lines in KAP1 and G9a KO mESCs, respectively (Figure 6A). Strikingly, the transcription start sites (TSSs) of nine of the sixteen 2C-specific genes upregulated in the G9a KO line overlap with or are within 5 kb of a MERVL/MT2 LTR. Similarly, analysis of the HP1α and HP1β KO RNA-seq datasets presented above revealed that 7% and 15% of 2C-specific genes are upregulated in these lines respectively, relative to the parent HM1 line. Taken together, 16% (42/264) of 2C genes are upregulated in one or both of the HP1 KO lines (Figure 6B). 2C-specific genes are significantly overrepresented among the genes upregulated in these KO mESCs, as only 1% of all genes in the blastomere RNA-seq data (of which there are 26,155) were identified as 2C-specific. Furthermore, of the nine 2C-specific genes upregulated in all four KO lines (Figure 6C), seven initiate transcription either within an MT2 LTR or within 5 kb of an MT2, MT2A or MT2C LTR, whereas only 4.5% (1387/30321) of all RefSeq genes are within 5 kb of an MT2, MT2A or MT2C annotated LTR.

Consistent with the observation that a subset of 2C genes are upregulated in these KO lines, genes shown previously to be expressed exclusively in early embryogenesis (confirmed to be 2C-specific in the analysis described above), including *Zfp352*[53], *Zscan4d*[54] and *Tdpoz3-4*[55] (Figure 6D), are upregulated in HP1α and HP1β KO mESCs as well as KAP1 and G9a KO mESCs (Figure 6E). Moreover, the top five genes showing the greatest difference in expression between the 2C stage and the oocyte and 8C stages: *Zfp352*, *Gm5039*, *Gm8994*, *B020004J07Rik* and *Dub1a*, also showed dramatic upregulation in HP1α and HP1β KO mESCs (Additional file 1: Figure S3A-B). Taken together, these observations reveal that, like KAP1 and G9a KO ESCs, HP1 KO mESCs show a significant increase in expression of a specific subset of 2C-specific genes, many of which are regulated by MERVL LTR promoters.

## Discussion

**Figure 7:**
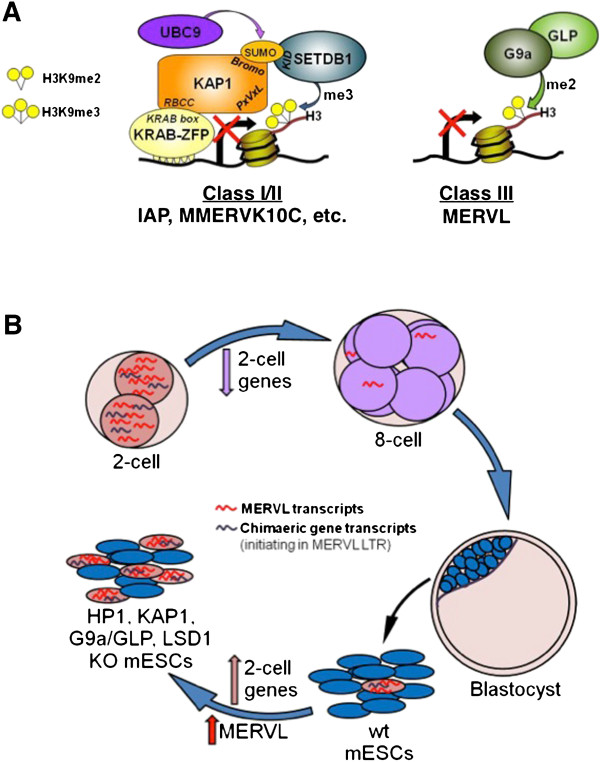
**Overview of transcriptional silencing mechanisms acting at different ERV families and the effect of chromatin factor depletion on mESC fate.** (**A**) The mechanism of transcriptional silencing of class I and II ERVs is distinct from that acting on the class III ERV MERVL. KAP1 is recruited to numerous class I and II ERVs, including IAP and MMERVK10C elements, via an interaction between the RBCC domain of KAP1 and the KRAB box of KRAB-ZFPs, which presumably bind directly to specific sequences within these ERVs. The SUMOylated bromodomain of KAP1 recruits SETDB1, which deposits the repressive H3K9me3 mark. MERVL elements in contrast, are bound by G9a and marked by H3K9me2 in a G9a-dependent manner. As these ERVs are upregulated in the absence of G9a or GLP, we propose that the G9a/GLP complex directly regulates MERVL expression via deposition of H3K9me2. (**B**) Depletion of specific chromatin factors, including KAP1 and HP1 proteins, may promote MERVL expression via indirect effects. MERVL transcripts and MERVL-driven genic transcripts are abundant at the 2C embryonic stage, but are rapidly depleted at subsequent stages, including the blastocyst, from which mESCs are derived. While a small fraction of wt mESCs continually enter a transient state associated with expression of multiple 2C-specific genes, the percentage of these cells increases dramatically in mESCs depleted of KAP1, G9a/GLP and LSD1 [11,12]. One or more of the 2C-specific genes commonly induced in these cells as well as in HP1α and HP1β KO mESCs may indirectly promote transcription of MERVL elements and MERVL LTR-driven genes.

MERVL elements are present in all placental mammals, suggesting that a common mammalian ancestor was colonized at least 70 million years ago. Several bursts of amplification have subsequently occurred in a number of lineages, including the mouse [56], which now harbors 600 to 700 full-length copies and approximately 37,000 solitary LTRs in the C57BL/6 genome. Intriguingly, a subset of MERVL LTRs may have been domesticated to serve as gene promoters specifically at the two-cell stage, when MERVL LTR promoters are highly transcribed [3,10]. While a small subset of sequences derived from ERVs may play a positive role by providing regulatory signals or encoding exapted proteins, proviral integration events are more likely to compromise host fitness. To counteract this threat, multiple mechanisms directed at various stages of the viral life cycle have evolved, including at the transcriptional level [57-60]. Our results reveal that distinct H3K9 methylation-based mechanisms of transcriptional silencing are used against different ERV families. At numerous class I and II ERVs, SETDB1 is recruited by KAP1 [44,61], which in turn interacts with one of multiple KRAB-zinc finger proteins that presumably recognize specific sequences within these ERVs to promote H3K9me3-mediated transcriptional silencing [44,61] (Figure 7A). Indeed, these parasitic elements are dramatically upregulated in KAP1- and SETDB1-deficient mESCs [24,28]. The relatively high levels of H3K9me3 [23,24], KAP1 and HP1 detected in the LTR and flanking genomic regions of class I and II ERVs, particularly IAP elements, is most consistent with a ‘spreading’ model, whereby deposition of H3K9me3 induces binding of HP1 proteins [35,36] and in turn KAP1 and SETDB1, which promotes deposition of H3K9me3 at neighboring nucleosomes in a process that occurs reiteratively. Curiously however, class I and II ERVs are not derepressed in mESCs deficient in HP1 proteins [25], leaving the role of HP1 proteins at class I and II ERVs in question.

Unlike class I and II ERVs, MERVL elements are neither bound by SETDB1 nor marked by H3K9me3, and deletion of SETDB1 does not dramatically induce expression of these class III ERVs. Furthermore, while deletion of KAP1, HP1α and HP1β promotes upregulation of MERVL elements, we did not detect significant enrichment of these factors at MERVL elements. While we cannot rule out the possibility that our ChIP assay is not sufficiently sensitive to detect binding of these chromatin factors to MERVL elements, we did detect significant enrichment of KAP1, HP1α and HP1β at IAP elements in the same experiments. A compelling explanation for the apparently distinct mechanisms underlying repression of these ERVs may be that MERVL elements are not bound by any of the over 300 KRAB-ZFPs encoded in the mouse genome [62,63]. While the striking diversity in KRAB-ZFPs was likely driven by selection for KRAB-ZFPs that recognize specific motifs within ERVs [64], MERVL-specific KRAB-ZFPs may simply not have evolved since the two bursts of MERVL retrotransposition in the mouse genome, which occurred 2 and 4 million years ago [56].

While this manuscript was under preparation, Macfarlan *et al*. reported that MERVL elements are upregulated in G9a KO mESCs [12], although the molecular basis of aberrant MERVL expression was not addressed. Here, we confirm this observation, and extend it to include mESCs deficient in the related KMTase GLP. Furthermore, we show that MERVL elements are bound by G9a and that maintenance of MERVL repression is dependent upon the catalytic activity of G9a but not GLP. As these elements are bound by G9a and marked by H3K9me2 in a G9a-dependent manner, we propose that the G9a/GLP complex acts directly at MERVL ERVs to maintain these elements in a silent state (Figure 7A). In contrast to IAP ERVs, MERVL ERVs show significantly higher levels of H3K9me2 than H3K9me3 enrichment. Taken together with the observation that HP1α and HP1β are detected at IAP but not MERVL ERVs, our results indicate that these H3K9 methylation ‘readers’ bind preferentially to H3K9me3 marked regions *in vivo*, though they are apparently not required for SETDB1-dependent silencing [25].

Although the molecular basis of H3K9me2-mediated transcriptional repression of MERVL elements remains to be determined, we propose that this pathway is particularly important in early embryogenesis. Consistent with this model, *G9a* and *Glp* mRNA levels are relatively low in the 2C embryo (Figure S3C) and H3K9me2 is depleted on the paternal genome at this stage [65-67], perhaps explaining why MERVL elements and zygotic genes driven by the MERVL promoters are expressed at the 2C stage and silenced shortly thereafter [3,10].

Intriguingly, depletion of several chromatin factors in mESCs including HP1α and HP1β (this study), G9a/GLP (this study and [12]), LSD1 [11], KAP1 [28], RYBP [68] and ZFP42 (REX1) [69], leads to upregulation of MERVL elements. Depletion of a subset of these factors, including KAP1, G9a, LSD1 and RYBP, also leads to derepression of genes highly expressed in preimplantation embryos, such as *Zfp352* and *Zscan4*[12,68]. Here, we show that the same is true for HP1α and HP1β KO mESCs. Derepression of MERVL elements in mESCs deficient in each of these chromatin factors may be due at least in part to an increase in the number of cells expressing 2C-specific genes, which may in turn stimulate MERVL expression (Figure 7B).

Complicating interpretation of these observations is the fact that a significant number of genes expressed specifically at the 2C stage initiate transcription within MERVL LTRs, such as *Zfp352*[53] and *Tdpoz4*[55], or are located within a few kb of a MERVL LTR [11,12]. Thus, it is not clear whether induction of a subset of the 2C-specific genes in the above mentioned KO mESCs induce MERVL expression as a ‘secondary effect’, or whether aberrant genic transcription of 2C genes in these KO lines results predominantly as a direct consequence of perturbation of MERVL silencing *per se*. These alternative explanations are not necessarily mutually exclusive.

## Conclusions

In summary, our results indicate that G9a and GLP play a direct role in silencing of MERVL ERVs and genes driven by MERVL LTR promoters via G9a-mediated deposition of H3K9me2, while the KAP1-interacting H3K9 KMTase SETDB1 is neither recruited to MERVL elements nor required for their repression. Conversely, the results presented here are consistent with the model that induction of MERVL expression following deletion of KAP1, HP1α and HP1β occurs primarily via indirect effects. Given that expression of MERVL elements, unlike other ERVs, is normally restricted to the 2C embryo, any perturbation of mESCs that induces a nuclear milieu permissive for expression of 2C-specific genes may indirectly induce expression of these ERVs as well as genes driven by MERVL promoters.

## Methods

### Cell culture, RNA isolation, qRT-PCR

Mouse ESC lines, including HP1α and HP1β KOs and their corresponding wt line HM1 [25] and G9a and GLP KOs and their corresponding wt line TT2 [70] were cultured in DMEM supplemented with 15% FBS (Thermo Scientific HyClone, Logan, UT, USA), 20 mM HEPES, 0.1 mM non-essential amino acids, 0.1 mM 2-mercaptoethanol, 100 U/ml penicillin, 0.05 mM streptomycin, leukemia-inhibitory factor and 2 mM L-glutamine on gelatinized plates. RNA was isolated using GenElute^™^ mRNA miniprep kit (Sigma-Aldrich, St Louis, MO, USA) and reverse transcribed using SuperScript III (Invitrogen, Carlsbad, CA, USA) as per the manufacturer’s instructions. Quantitative RT-PCR was carried out using SsoFAST^™^ EvaGreen Supermix (Bio-Rad, Hercules, CA, USA) on StepOne^™^ Software v2.1 (Applied Biosystems, Foster City, CA, USA). Data are presented as mean +/- standard deviations of three technical replicates. Primer efficiencies were 95 to 105%. Dissociation curve analysis was performed after the end of the PCR to confirm the presence of a single and specific product. Corresponding ERV primers detect 519 MERVL elements, 202 MMERVK10C elements and 638 IAPEz elements. Primer sequences are listed in Additional file 3.

### RNAi

For RNA collection, 7,000 mESCs per well of a 96-well plate were plated into antibiotic-free ES medium the day before transfection. Transfection was performed according to the manufacturer’s protocol, using 100 nM of each siRNA (Dharmacon siGENOME SMARTpool) and 0.4 μl DharmaFECT 1 reagent per well (Thermo Scientific Dharmacon, Lafayette, CO, USA). The first day after transfection, a fraction of cells was transferred into a 12-well plate into antibiotic-free ES medium, and the transfection was repeated on the third day. The following day (approximately 30 h) after the second KD, most of the cells were collected for RNA for confirmation of KD efficiency (day 1 after the second KD), and the rest were plated onto 3.5-cm dishes for expansion and collection of RNA to monitor ERV derepression on day 4 after the second KD. For ChIP on day 4 after the second KD, the same steps were performed accounting for the increased volumes, with the cells plated onto a 12-well plate for the first transfection, transferred onto two 6 cm dishes for the second transfection and onto 4 to 6 × 10 cm dishes for collection on day 4 post second transfection, with approximately 2 × 10^5^ cells saved at day 1 (30 h) for the RNA analysis of KD efficiency.

### Native ChIP (N-ChIP) and cross-linked ChIP

For N-ChIP, 1 × 10^7^ ES cells for each cell line were resuspended in douncing buffer (10 mM Tris–HCl pH 7.5, 4 mM MgCl_2_, 1 mM CaCl_2_) and homogenized through a 25 G5/8 needle syringe for 20 repetitions. Following addition of 1.25 μl (50 U/μl) of MNase (Worthington Biochemicals Corp., Lakewood Township, NJ, USA), the sample was incubated at 37°C for 7 min. The reaction was quenched with 0.5 M EDTA and incubated on ice for 5 min. 1 ml of hypotonic buffer (0.2 mM EDTA pH 8.0, 0.1 mM benzamidine, 0.1 mM PMSF, 1.5 mM DTT) was added and the sample incubated on ice for 1 h. Cellular debris was pelleted and the supernatant was recovered. Protein A (Millipore, 16–156) and G Sepharose (Millipore, 16–266) (Millipore, Billerica, MA, USA) beads were blocked with single-stranded salmon sperm DNA and BSA, washed and resuspended in IP buffer (10 mM Tris–HCl (pH 8.0), 1% Triton X-100, 0.10% deoxycholate, 0.10% SDS, 90 mM NaCl, 2 mM EDTA). Blocked protein A/G beads were added to the digested chromatin fractions and rotated at 4°C for 2 h to preclear chromatin. An aliquot of a 100 μl of precleared chromatin was purified by phenol-chloroform extraction, and DNA fragment sizes were analyzed and confirmed to correspond to 1 to 3 nucleosome fragments. Chromatin was subdivided into aliquots for each IP. Antibodies specific for unmodified H3 (5 μl, Sigma-Aldrich H9289), H3K9me1 (5 ul, Abcam ab8896;), H3K9me2 (5 μl, Abcam ab1220) (Abcam, Cambridge, UK), H3K9me3 (5 μl, Active Motif 39161) (Active Motif, Carlsbad, CA, USA), and control IgG (1 μl, Sigma-Aldrich I8140) were added to each tube and rotated at 4°C for 1 hour. The antibody-protein-DNA complex was precipitated by adding 20 μl of the blocked protein A/G beads and rotated at 4°C overnight (O/N). The complex was washed and eluted; IP’d material was purified using the QIAquick PCR Purification Kit (Qiagen, Venlo, Netherlands).

Cross-link ChIP for HP1 proteins was performed as described by Metivier *et al*. [71]. Briefly, 2 × 10^7^ ES cells were harvested. Chromatin was cross-linked in 10 ml of PBS + 1% formaldehyde for 10 min at room temperature (RT). Reaction was quenched with 0.125 M glycine and incubated at RT for 5 min. Cells were washed two times with PBS and resuspended in 1 ml of collection buffer. The solution was incubated on ice for 10 min, then for 10 min at 30°C. Cells were lysed by vortexing pellets sequentially in 1 ml buffer A, 1 ml buffer B, followed by incubation of cell pellets at RT in 1 ml of lysis buffer for 10 min. Chromatin was sonicated using the Diagenode Bioruptor (Diagenode, Liege, Belgium) at high setting for 18 min at intervals of 30 sec ‘on’ and 30 sec ‘off’ to achieve fragment lengths of 250 to 500 bp. For each IP, 2 × 10^6^ cell-equivalents of precleared chromatin were used and diluted 2.5 times in IP buffer. Antibodies specific for HP1α (10 μl, academic, S. Smale), HP1β (5 μl, NEB, HP1beta (D2F2) XP™ rabbit mAb 8676) and control IgG (1 μl, Sigma-Aldrich I8140) were added into each IP and rotated at 4°C O/N. To each tube, 40 ul of precleared beads were added, and tubes were rotated at 4°C for 2 h. The complex was washed and eluted off the beads. The cross-links were reversed O/N at 65°C, and the DNA was treated with Proteinase K and RNase A. DNA was purified using the Qiaquick PCR Purification Kit (Qiagen) and analyzed by qPCR with respect to input using SsoFAST™ EvaGreen Supermix (Bio-Rad). Primer sequences are listed in Additional file 3.

Cross-linked ChIP for G9a and KAP1 were performed similarly with several modifications. Approximately 5 × 10^6^ TT2 or G9a KO mESCs were harvested cross-linked in 0.75% formaldehyde in PBS for 10 min, quenched with glycine, and lysed in ice-cold radioimmunoprecipitation assay buffer (RIPA) (50 mM Tris pH 8.0, 150 mM NaCl, 1% NP-40, 0.25% sodium deoxycholate, 0.1% SDS). Chromatin was sonicated with the Bioruptor on 30 sec ‘on’ and 30 sec ‘off’ to achieve 200 to 600 bp fragment size for each sample. The samples were divided into three equal aliquots and incubated O/N at 4°C with 4 μg of purified mouse IgG (Sigma-Aldrich), mouse monoclonal anti-G9a (R&D Systems PP-A8620-00) (R&D Systems, Minneapolis, MN, USA), or mouse monoclonal anti-KAP1 (Abcam Ab22553). Samples were precipitated with 30 μl protein-G Dynabeads (Invitrogen), washed three times with ice-cold RIPA buffer and eluted by shaking in 0.1 M NaHCO_3_, 1% SDS, 20 mM DTT for 15 min. Cross-links were reversed by heating samples at 95°C for 5 min in the presence of 300 mM NaCl. DNA was RNase A-treated, purified and qPCR was performed as described above. Primers used or ChIP-qPCR analyses detect: 638 IAP ERVs, 519 MERV-L ERVs, 202 MMERVK10C ERVs, 637 MERV-L *pol* internal regions, as determined by *in silico* PCR on the UCSC genome browser.

### RNA-seq, data normalization and Z-score calculation

RNA-seq libraries were constructed from mRNA as described in Morin *et al*. [72] from 10 μg of DNaseI-treated total RNA, and 75 bp paired-end sequencing was performed on an Illumina Genome Analyzer following the recommended protocol (Illumina Inc., Hayward, CA, USA). Sequence reads were aligned to the mouse reference genome (mm9) using BWA v0.5.9 [73] with Smith-Waterman alignment disabled and annotated exon-exon junctions compiled from Ensembl [74], RefSeq [75] and UCSC [76] (downloaded from http://genome.ucsc.edu on 17 August 2011). To quantify expression levels and the strength of KAP1 and SETDB1 marks, we calculated reads per kilobase per million mapped reads (RPKM) [77,78] for genomic regions of interest. For pair-wise sample comparisons, an empirical Z-score was calculated assuming the distribution of RPKMs for each sample followed a Poisson model:

$$Z-\mathrm{score}=\left(\mathrm{RPK}M_{A}-\mathrm{RPK}M_{B}\right)/\sqrt{\left(\mathrm{RPK}M_{A}-r_{\mathrm{AB}}\mathrm{RPK}M_{B}\right)}$$

where RPKM_A_ and RPKM_B_ are RPKMs in the region of interest of A and B samples respectively, and r_AB_ = N_A_/N_B_, where *Nx* is the total number of aligned reads used for normalization.

### KAP1 ChIP-seq data analysis

In order to compare the coverage of KAP1 and SETDB1 among all families of ERVs and generate the average density of KAP1 in the genomic regions flanking ERVs, we mined the published KAP1 ChIP-seq data set [46]. Sequence reads for KAP1 were remapped to mm9 (NCBI 37) using BWA v0.5.9 [79] and default parameters. Reads having identical coordinates were collapsed into a single read, and reads with mapQ > =10 passed to FindPeaks 3.1 [80] (with a fixed directional read extension of 300 bp) for generation of an unthresholded coverage WIG file to be visualized in the UCSC genome browser [81]. This coverage file was used to calculate KAP1 RPKM values for various regions of interest. Subsequently, we identified all enriched regions with a peak-height ≥10 and generated a thresholded coverage WIG file for KAP1, using FindPeaks. This WIG file was used to generate the profiles at the genomic regions flanking ERVs.

The KAP1 profile was generated at the genomic flanks of intact elements (flanked by two identical LTRs), which satisfied the length criteria, for three ERV families: MMERVK10C, IAPEz and MERVL. The mean density of KAP1 for each family was calculated for 50 bp bins within 6 kb distance at 5′ and 3′ flanks of elements by agglomerating the coverage inside the bins for all elements of one family and dividing this number by the number of elements and total number of aligned reads in the KAP1 IP.

### Detection of chimeric transcripts

Chimeric transcripts containing ERV and genic sequences were identified by exploiting the genomic locations of paired-end reads. Mate-pair reads separated by more than one standard deviation from the mean fragment size were identified, and those mate-pairs containing one read in an ERV located upstream of the first exon of a gene, and the other read in an annotated genic exon of that gene, were enumerated. The number of chimeric mate-pairs was calculated for each chimeric transcript and the transcripts with the number of chimeric mate-pairs >5 were scored as valid transcripts.

### Immunoprecipitation and Western blotting

Whole-cell extracts were prepared by lysing cells in modified RIPA buffer (50 mM Tris pH 8.0, 150 mM NaCl, 1% NP-40, 0.25% deoxycholate, 0.1% SDS). Nuclear extracts were prepared as previously described [82] except nuclei were extracted for 30 min in 20 mM HEPES pH 7.9, 500 mM KCl, 1.5 mM MgCl2, 25% glycerol. Protein concentrations were determined by Bradford assay (Bio-Rad) using BSA as a standard. For immunoprecipitation, 200 μg of nuclear extract was diluted to approximately 170 mM KCl incubated with 1 μg of mouse monoclonal anti-KAP1 (Abcam ab22553), mouse monoclonal anti-G9a (R&D Systems PP-A8620-00), or mouse IgG (Sigma-Aldrich) and samples were rotated overnight at 4°C. Complexes were precipitated with protein G-Dynabeads (Invitrogen), washed three times with ice-cold wash buffer (20 mM HEPES pH 7.9, 200 mM KCl, 1% NP-40, 10% glycerol) and eluted by boiling beads in SDS-PAGE sample buffer containing DTT. For Western blot analysis, protein extracts or immunoprecipitated samples were separated on 7.5% or 10% SDS-PAGE gels, transferred to nitrocellulose membranes, blocked with 4% skim milk in Tris-buffered saline (TBS: 20 mM Tris–HCl pH 7.5, 100 mM NaCl) and probed overnight at 4°C with primary antibodies diluted in TBS containing 0.1% Tween-20 (TBS-T): 1:5000 mouse anti-KAP1 (Abcam), 1:1000 mouse anti-G9a (R&D Systems), 1:1000 mouse anti-GLP (R&D Systems PP-B0422-00), 1:2000 rabbit anti-LSD1 (Abcam ab17721), 1:500 rabbit anti-HP1β (Cell Signaling 2613) (Cell Signaling Technology, Danvers, MA, USA) and 1:500 rabbit anti-Pol II large subunit (Santa Cruz Biotechnology sc-899) (Santa Cruz Biotechnology, Santa Cruz, CA, USA). Blots were subsequently washed in TBS-T, incubated in IRDye-conjugated secondary antibodies diluted 1:20,000 in 2% milk in TBS-T washed again in TBS-T and scanned on the Odyssey infrared imaging system (LI-COR Biosciences, Lincoln, ME, USA).

### Availability of supporting data

The data sets supporting the results of this article are available in NCBI's Gene Expression Omnibus repository [83], GEO Series accession number GSE47370 (http://www.ncbi.nlm.nih.gov/geo/query/acc.cgi?acc=GSE47370).

## Competing interests

The authors declare that they have no competing interests.

## Authors’ contributions

IM performed most experiments, PT performed qRT-PCR of *Zfp352*, ChIP-qPCR of G9a and KAP1 and Western blotting, PG performed ChIP-qPCR of HP1s, MK performed bioinformatics analyses, with support from SJ, PS contributed the HP1 KO lines, IM and ML designed the study and wrote the manuscript. All authors read and approved the final manuscript.

## Supplementary Material

Additional file 1 Figures S1-S3 and supporting figure legends. Click here for file

Additional file 2 **Table of annotated genic transcripts initiating in a MERV-L LTR.** All protein-coding genes that initiate in an annotated MERV-L/MT2 LTR in both RefSeq and ENSEMBL databases were identified and cumulative RPKM values at annotated exons for each gene were quantified for KAP1 [28] and G9a [12] KO ESCs, as well as their wt parent lines using previously published RNA-seq datasets. Z-score and fold-change values were calculated based on these RPKM values. RPKM, Z-score and fold-change values were also generated from RNA-seq data generated from HP1α and HP1β KO lines and their wt parent line HM1. Click here for file

Additional file 3 Table of primer sequences used in this study. Click here for file
