# Supplementary material for: Distinct roles of KAP1, HP1 and G9a/GLP in silencing of the two-cell-specific retrotransposon MERVL in mouse ES cells
Source: Epigenetics Chromatin. 2013 Jun 4;6:15. doi: 10.1186/1756-8935-6-15 (PMC3682905; doi:10.1186/1756-8935-6-15)
Supplement: Additional file 1 — Figures S1-S3 and supporting figure legends. [file 1756-8935-6-15-S1.pdf]

Additional file 1

Figure S1: (A) Western blot analysis of G9a, GLP, LSD1, KAP1 and RNA pol II (Pol II) in TT2 wt, G9a KO and GLP KO mESCs reveals that KAP1 and LSD1 show no change in these KO lines. **(B)** Antibodies specific for KAP1 and G9a, or IgG as a control, were used in co-IP experiments on mESC nuclear extracts. Approximately 50% of IP'd material was analyzed by Western blotting using KAP1-, G9a-, GLP- and HP1β-specific antibodies. **(C)** Upper panel: a UCSC genome browser screen shot of the *Zfp352* locus (mm9; chr4:89,883,794-89,894,095) is shown, along with the Repeatmasker track for this region. Note that the MT2B LTR (light grey box) encompasses annotated exon 1. RT-PCR primers used in the experiment depicted below are shown as horizontal arrows. Lower panel: qRT-PCR analysis of *Zfp352* in *Glp* KD mESCs, reveals dramatic upregulation of this gene in the KD sample relative to the scrambled siRNA control. The mean expression level (+/-SD) is shown for three technical replicates (normalized to *Gapdh*).

Figure S2: (A) Z-scores of expression (RPKM) values for all LTR elements in HP1β KO and KAP1 KO mESCs, relative to their cognate wt parent lines, are shown (KAP1 RNA-seq data from reference [28]). **(B)** Z-score values for individual full-length MERVL elements in HP1α and HP1β KO mESCs, relative to the wt HM1 parent line, are shown. **(C)** Pie charts show the fractions of LTR-chimaeric transcripts of each ERV class in HM1 wt and HP1 KO mESC lines, as determined by analysis of paired-end RNA-seq data. The total number of chimaeric genes driven by MERVL LTRs is given in parentheses. **(D-E)** ChIP was conducted on control siRNA (Scram) or *Kap1* siRNA transfected mESCs, using an HP1α-specific antibody and IgG as a negative control. A high level of enrichment was detected by qPCR at both the *Mest* and IAPEz promoter regions, relative to the IgG control. Mean enrichment values for three technical replicates, as a percentage of the input chromatin, are shown. Error bars represent the standard deviation.

Figure S3: (A-B) Expression (RPKM) values of the top five two-cell specific genes in oocytes and early embryos, based on meta-analysis of published RNA-seq data [52], are shown. **(B)** Meta-analysis of published RNA-seq data for G9a [12] and KAP1 KO ESCs [28] as well as our own RNA-seq data for HP1α and HP1β KO ESCs, reveals that all are significantly upregulated in the HP1-deficient lines, as measured by Z-scores. **(C)** Expression (RPKM) values from published RNA-seq data for oocytes and early embryos are shown for *Setdb1, Kap1, G9a* and *Glp* [52].

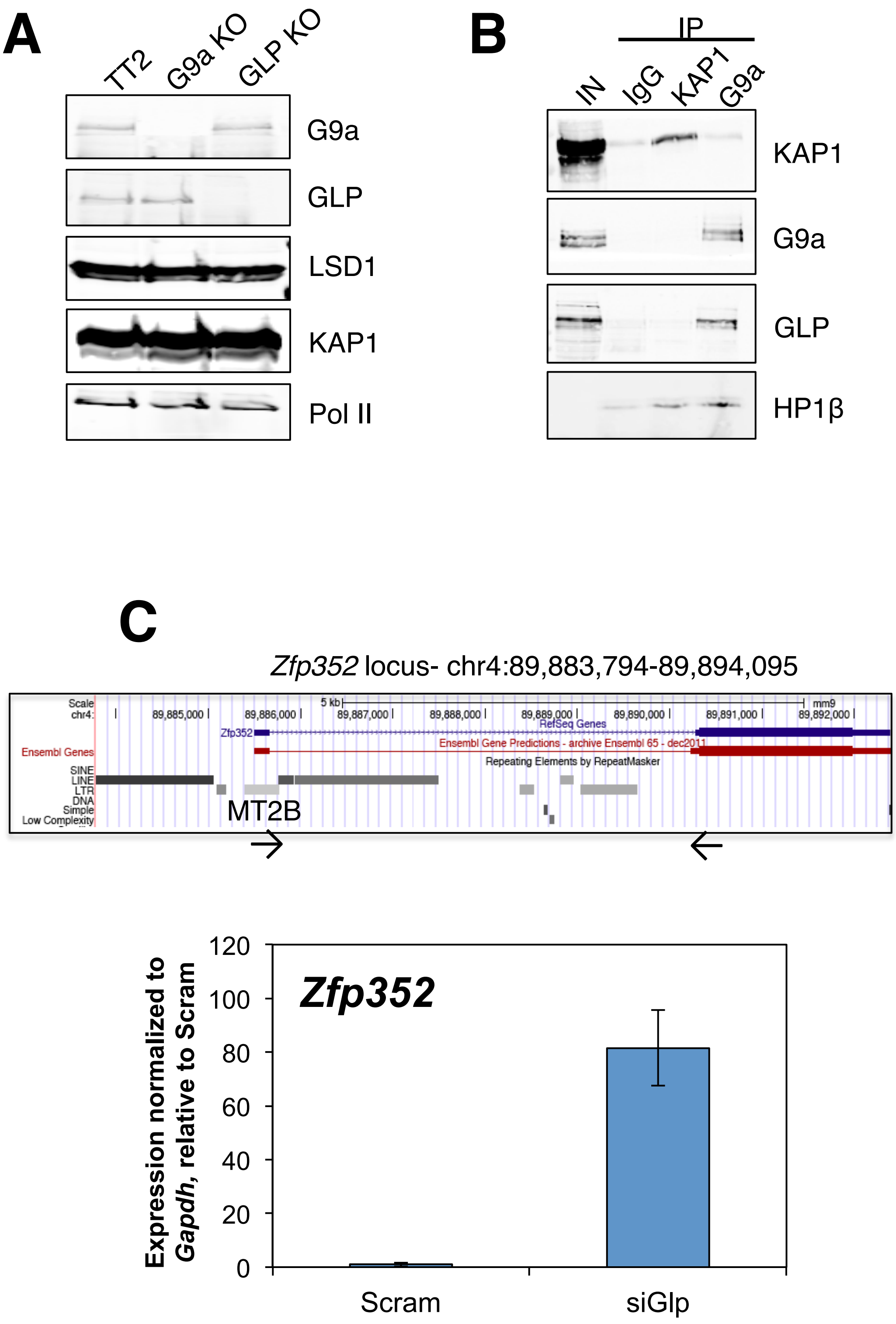

Supplementary Figure S1. Maksakova et al. 2012

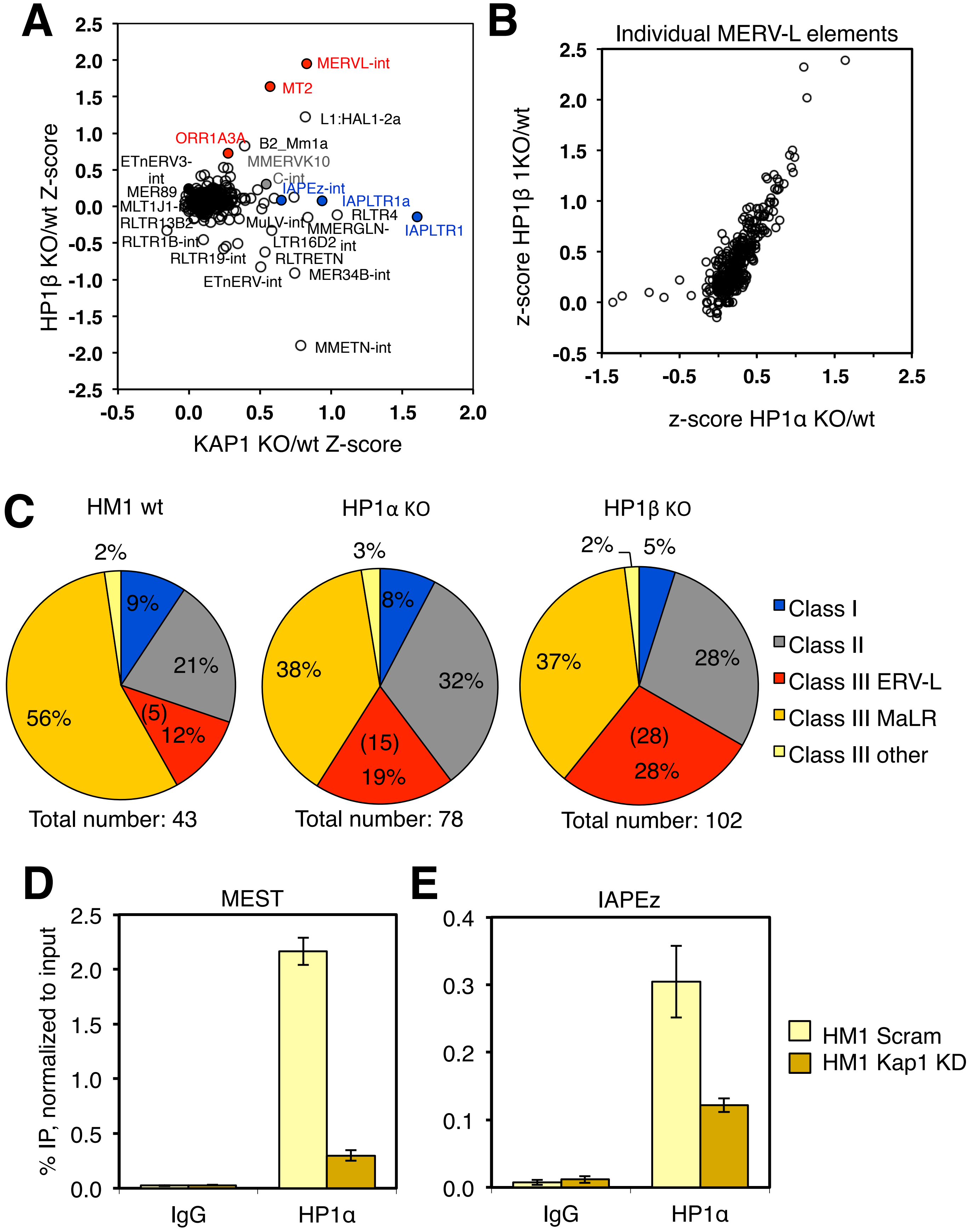

Supplementary Figure S2. Maksakova et al. 2012

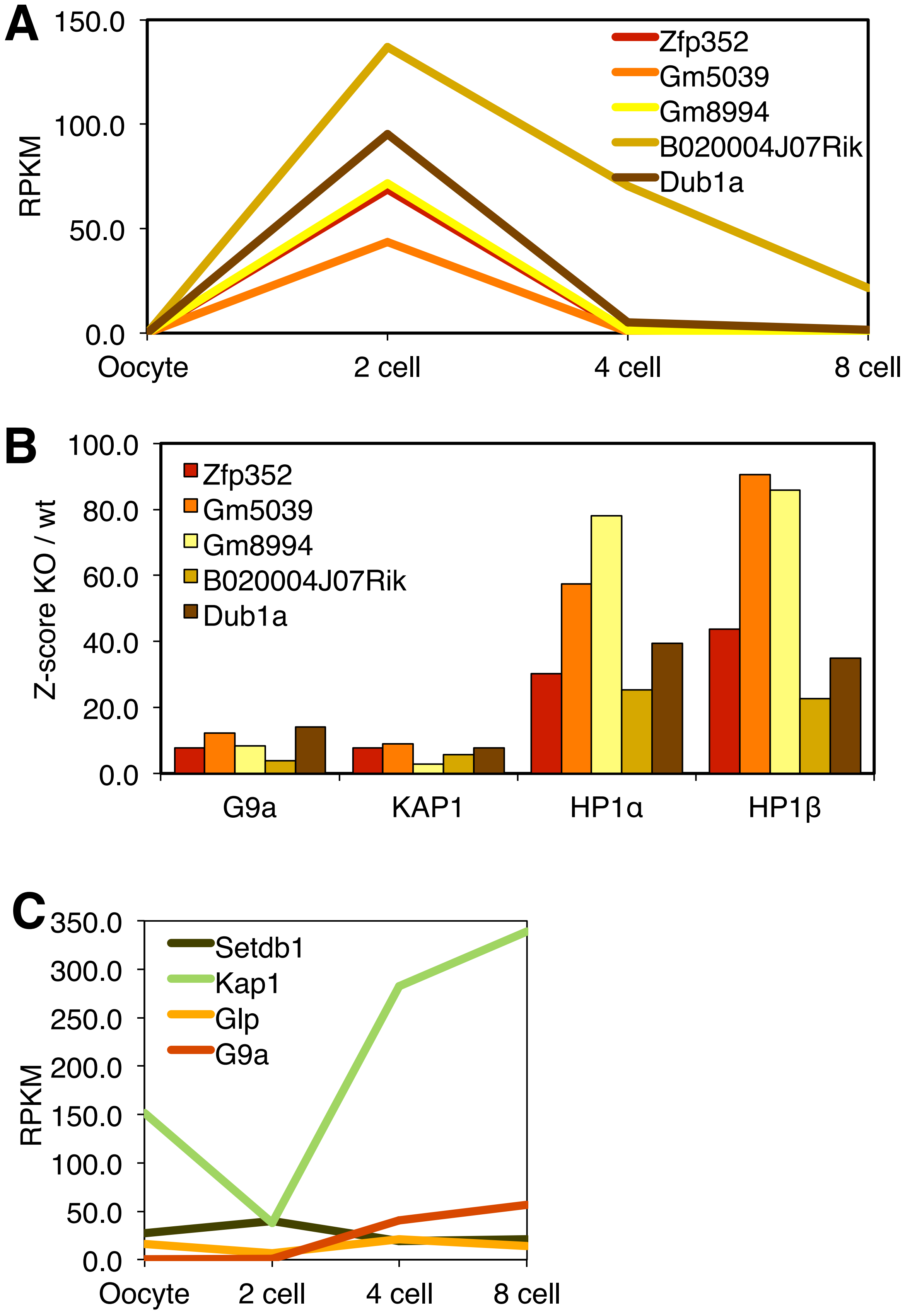

Supplementary Figure S3. Maksakova et al. 2012
